# X-Ray Phase-Contrast Imaging with Three 2D Gratings

**DOI:** 10.1155/2008/827152

**Published:** 2008-03-24

**Authors:** Ming Jiang, Christopher Lee Wyatt, Ge Wang

**Affiliations:** ^1^Laboratory of Mathematics and Applied Mathematics (LMAM), School of Mathematical Sciences, Peking University, Beijing 100871, China; ^2^Biomedical Imaging Division, VT-WFU School of Biomedical Engineering and Sciences, Virginia Polytechnic Institute and State University, Blacksburg, VA 24061, USA; ^3^Electrical and Computer Engineering Department, Virginia Polytechnic Institute and State University, Blacksburg, VA 24061, USA

## Abstract

X-ray imaging is of paramount importance for clinical and preclinical imaging but it is fundamentally restricted by the attenuation-based contrast mechanism, which has remained essentially the same since Roentgen's discovery a century ago. Recently, based on the Talbot effect, groundbreaking work was reported using 1D gratings for X-ray phase-contrast imaging with a hospital-grade X-ray tube instead of a synchrotron or microfocused source. In this paper, we report an extension using 2D gratings that reduces the imaging time and increases the accuracy and robustness of phase retrieval compared to current grating-based phase-contrast techniques. Feasibility is demonstrated via numerical simulation.

## 1. INTRODUCTION

Since its introduction in 1973, X-ray computed tomography (CT) has revolutionized medical imaging and become a cornerstone of modern radiology. Improved resolution and reduced dose are two critical requirements in biomedical applications and remain the focus of CT research and development. The rapid development of small animal models of various human diseases has also generated the need for preclinical imaging. Spurred by the refinements of CCD cameras and microfocus X-ray tubes in the 1990s, a number of micro-CT systems were constructed, reaching image resolutions between 10 and 100*μ*m. These scanners, while producing high-resolution images, do not provide high-contrast or low-dose imaging of in vivo processes in small animal models. Most importantly, many normal and diseased tissues such as cancers display poor image contrast on CT/micro-CT images.

Current X-ray CT techniques generate contrast from differences in attenuation. Hence, weakly absorbing tissues display poor contrast [1]. The X-ray absorption coefficient is roughly proportional to the fourth power of the atomic number *Z*, apart from the jumps at absorption edges [2]. Therefore, soft tissues are difficult to image in terms of only the magnitude of the X-ray beam. However, X-ray phase-contrast imaging uses the diffraction properties of X-rays and reveals significant differences indistinguishable in an attenuation coefficient distribution. The wave propagation of X-rays is characterized macroscopically by the complex refraction index of the underlying medium. The refraction index *n* of soft biological tissues is approximately 1, namely, *n* = 1 − *δ* + *iβ* where *β* and *δ* quantify the intensity attenuation and phase shift, respectively. At X-ray energies of 15–25 keV, *δ* is about three orders of magnitude greater than *β*, depending on the X-ray wavelength and electron density. Thus, phase-contrast imaging enjoys a sensitivity to soft-tissue 1000 times greater than that with attenuation-based imaging [3]. Moreover, because the cross-section of the X-ray phase shift varies slower than the attenuation counterpart at higher energies, X-ray phase-contrast imaging can be performed at higher energies, further reducing radiation dose. More importantly, the phase-contrast mechanism is potentially a new design criterion for contrast agents, including those that can be functionalized for molecular imaging.

The use of phase shift as an X-ray contrast mechanism has generated considerable interest over the past decade [1]. X-ray phase-contrast imaging can be implemented using interferometry or diffractometry approaches which measure the first order derivative of the phase shift, or inline holography approaches which measure the X-ray intensity. Because traditional interferometry and diffractometry require monochromatic X-rays and high-precision crystals, they are restricted to synchrotron radiation. The inline method is feasible with a polychromatic X-ray tube, and thus more practical.

Gabor originally proposed the inline holography approach in 1948, for which he was awarded the Nobel Prize. In 2000, Takeda et al. demonstrated how phase-contrast imaging captures structures in carcinoma with 30 *μ*m resolution, and observed that the X-ray energy for phase-contrast imaging can be increased from 17.7 keV to 30 keV. Wilkins et al. developed inline holography techniques with a polychromatic microfocus X-ray source [4]. Wu et al. generalized the theory and improved the techniques along this direction [5, 6]. In these kinds of systems, the phase retrieval is done by either free-space propagation or grating-based shearing interferometer. The advantage of using the interferometer is both sensitivity in the phase shift measurement and insensitivity to mechanical drift or vibration. However, such systems are limited by the spatial coherence requirement. While synchrotrons are expensive, microfocus (< 100 *μ*m) X-ray tubes require a low-duty cycle due to limited spot size and resultant anode heating.

Recently, grating-based phase-contrast X-ray interferometry has been developed with very promising results [7–11]. The working principle is nearly the same as that of Talbot interferometry [12–14] in the visible light regime. Such a system uses a conventional (noncoherent) X-ray tube and gratings to overcome the well-known problem of spatial and temporal incoherence [11, 15]. Specifically, their approach uses three 1D gratings to produce coherent wavelets from a hospital X-ray tube, construct interference patterns at an appropriate distance, and detect phase changes from wave-fronts distorted by an object. Thus, quantitative phase-contrast imaging becomes possible in the case of centimeter-sized objects. This grating-based interferometric approach has advantages over other X-ray phase-contrast imaging techniques because it allows the cone-beam imaging geometry, large field of view, and efficient use of polychromatic low-brilliance sources [16]. Since the total phase shift is an integral over the X-ray path length that is nearly straight, it generates projections of the refractive index that can be inverted using X-ray CT techniques [8]. For example, X-ray phase-contrast tomography was first performed using crystal X-ray interferometry in combination with phase-shifting interferometry [1, 17].

The developments outlined above represent both an outstanding opportunity and major challenges in the field for practical widespread use. In this paper, we develop the theory, methods, and top-level system design so that a conventional hospital-grade X-ray tube may be used in a practical way for phase-contrast imaging of small animals. Our general hypothesis is that 2D grating-based phase-contrast imaging techniques can be developed to produce more informative projective, and tomographic images of biomedical interest than current techniques.

The rest of the paper is organized as follows. In the second section, we will propose a 2D grating-based phase-contrast imaging system and formulate the forward imaging model. In the third section, we will describe our numerical simulations and report representative results. Finally, we conclude by discussing relevant issues.

## 2. SYSTEM AND METHOD

### 2.1.Top-level design of a 2D grating-based system

Our proposed 2D grating-based system architecture requires three gratings: the source grating G0, the phase grating G1, and analyzer grating G2, which are all aligned along the principal optical axis. The source grating G0 is placed right behind an X-ray source, acts as a set of apertures to form a 2D array of virtual sources. The object under imaging is placed in front of the phase grating G1. The phase grating G1 is placed at a distance *L* from the source grating G0. The phase grating G1 induces a periodic spatial modulation in either amplitude or phase of the resultant wave front after X-ray penetrates the phase object. The phase and analyzer gratings G1 and G2 are of the same periodicity *p_x_* and *p_y_* in the *x* - and *y*- directions, respectively, and must be separated at a Talbot distance *d* The distance *d* can also be one of the fractional Talbot distances. However, a smaller *d* is preferable in practice. We choose *d* to be the first Talbot distance *Z_T_* in (8) in this study, though there are alternative choices at a smaller fractional Talbot distances.

In this setup, each virtual source from the source grating G0 is incoherent with respect to the others, and small enough to create a fringe pattern with good visibility in the image plane after the analyzer grating G2. As the source mask G0 can contain a large number of individual apertures, each creating a sufficiently coherent virtual point source, standard X-ray generators with source sizes of more than a square millimetre can be used efficiently [11]. This key observation implies that hospital X-ray tubes can be used for phase-contrast imaging [11, 15, 16, 18]. To ensure that each virtual source contributes constructively to the fringe patterns at appropriate distances downstream, by a geometrical argument as in [11, 15, 16, 18], the periodicity of the source grating G0 is required to be

(1) $$p_{0,x}=\frac{L}{d}p_{x},\;\;p_{0,y}=\frac{L}{d}p_{y}.$$

The longitudinal or lateral periodicity of the fringes does not depend on the wavelength of the radiation [19]. Hence the setup is achromatic, allowing the use of a broadband source. These fringe patterns can be detected using a CCD camera. The phase-stepping approach [20–22] can be adapted to retrieve the gradients of the object phase shift, from which it is possible to reconstruct the phase profile of the object.

The gratings are usually made of gold and/or silicon. The thickness of gold pillars of several tens of microns is considered sufficient to block X-rays effectively. Roughly speaking, 10 *μ*m is needed at 20 keV, 30 *μ*m at 30 keV, and 60 *μ*m at 40 keV [23]. Furthermore, the pitch of the grating must be about the spatial coherent length of primary X-rays, which is on the order of microns. Such an X-ray amplitude grating can be fabricated by lithography and in the case of the G0 and G2 gratings, gold electroplating, followed by coating in epoxy and bonding to a frame for mechanical stability [23].

### 2.2.Theory of 2D Talbot interferometry

In this section, we present the mathematical theory for the setup in the previous section.

#### 2.2.1.Talbot effect under plane wave illumination

Talbot effect of 2D grating was first analyzed in [24]. The treatment was refined further in the following years. We consider the case of a 2D grating G1 illuminated coherently by a unit-amplitude plane-wave X-rays exp (*ikz*) of wavelength *λ* where *k* = 2*π*/*λ* is the wave number, though the case of Gaussian beams can also be investigated [25]. The optical axis is parallel to the *z*-axis. The 2D grating G1 on the *xy*-plane at *z* = 0 is of periods *p_x_* and *p_y_* in the *x*- and *y*, respectively. The complex transmittance function of G1 is given by the following Fourier series:

(2) $$T_{1}\left(x,y\right)=\underset{m,n}{\sum }a_{m,n}\exp\left(\frac{i2\pi mx}{p_{x}}\right)\exp(\frac{i2\pi ny}{p_{y}}).$$

We assume that gratings are infinitely extended in this preliminary study. Under a paraxial approximation, the wave field at a distance *z* behind the grating G1 is, by the Fresnel-Kirchhoff diffraction formula [25–27],

(3) $$E\left(x,y,z\right)=\exp\left(ikz\right)\underset{m,n}{\sum }\beta_{m,n}\left(z\right)\exp\left(\frac{i2\pi mx}{p_{x}}+\frac{i2\pi ny}{p_{y}}\right),$$

where

(4) $$\beta_{m,n}\left(z\right)=a_{m,n}\exp\left(-\frac{i\pi \lambda m^{2}z}{p_{x}^{2}}-\frac{i\pi \lambda n^{2}z}{p_{y}^{2}}\right).$$

The intensity of the wave field is given by

(5) $$\begin{array}{ll} I\left(x,y,z\right) & ={\left|E\left(x,y,z\right)\right|}^{2}\\ & =\underset{m,n}{\sum }\rho_{m,n}\left(z\right)\exp\left(\frac{i2\pi mx}{p_{x}}+\frac{i2\pi ny}{p_{y}}\right), \end{array}$$

where

(6) $$\rho_{m,n}\left(z\right)=\underset{m',n'}{\sum }\beta_{m+m',n+n'}\left(z\right)\overset{¯}{\beta_{m',n'}\left(z\right)}$$

Let the periods *p_x_* and *p_y_* be of a rational ratio in the following sense:

(7) $$p_{x}=Mp,\;\;p_{y}=Np,$$

for some positive numbers *M*, *N*, and *p*, where *M* and *N* are integers. Let

(8) $$Z_{T}=\frac{2M^{2}N^{2}p^{2}}{\lambda }.$$

Then we have

(9) $$\beta_{m,n}\left(z\right)=a_{m,n}\exp(-2\pi iN^{2}m^{2}\frac{z}{Z_{T}}-2\pi iM^{2}n^{2}\frac{z}{Z_{T}}).$$

It follows that

(10) $$\beta_{m,n}\left(z+sZ_{T}\right)=\beta_{m,n}(z),$$

for any integer *s* ∈ ℤ. Hence, the wave field is longitudinally periodic in the *z*-direction.

This is the Talbot effect discovered in [12]. The same complex amplitude of the wave field as that of the complex transmission function of the grating is generated at *z* = *sZ_T_*, which is referred to as the Talbot images or self-images in [25, 28]. At other distances such as

(11) $$D_{u,v}=\frac{v}{u}Z_{T},\;u=1,2,\ldots ,v=1,2,\ldots ,$$

The wave field can be expressed as a superposition of laterally shifted replicas of the exit wave field right after the grating [26]. It is not possible to find reassembled images at every fractional Talbot distance *D_u,v_*. It depends on the fraction *v*/*u* and the shape of the grating groove [28].

Some examples are as follows. For *u* = 2 and *v* = 1,

(12) $$E\left(x,y,{z|}_{z=D_{u,v}}\right)=\exp\left(ikz\right)T_{1}(x+\frac{MNp}{2},y+\frac{MNp}{2});$$

for *u* = 4 and *v* = 1,

(13) $$\begin{array}{ll} & E\left(x,y,{z|}_{z=D_{u,v}}\right)\\ & =\frac{\exp\left(ikz\right)}{2i}[T_{1}\left(x,y\right)-T_{1}(x+\frac{MNp}{2},y+\frac{MNp}{2})], \end{array}$$

which is quite different from the 1D grating case [26, 29]. 

#### 2.2.2.Phase stepping technique

When a phase object that causes a phase shift Φ(*x, y*) is placed in front of the grating G1, under a stationary phase approximation, the wave field at a distance *z* behind the grating G1 is

(14) $$\begin{array}{ll} E_{S}\left(x,y,z\right) & \approx \exp\left(ikz\right)\underset{m,n}{\sum }\beta_{m,n}\left(z\right)\\ & \;\times \exp(\frac{2\pi imx}{p_{x}}+\frac{2\pi iny}{p_{y}}\\ & \;\;\;\;\;+i\Phi \left(x-\frac{m\lambda z}{p_{x}},y-\frac{n\lambda z}{p_{y}}y\right)). \end{array}$$

Because

(15) $$\begin{array}{ll} \Phi \left(x-\frac{m\lambda z}{p_{x}},y-\frac{n\lambda z}{p_{y}}\right) & \approx \Phi \left(x,y\right)-\frac{m\lambda z}{p_{x}}\frac{\partial \Phi }{\partial x}\left(x,y\right)\\ & \;-\frac{n\lambda z}{p_{y}}\frac{\partial \Phi }{\partial x}\left(x,y\right), \end{array}$$

we obtain

(16) $$\begin{array}{ll} E_{S}\left(x,y,z\right) & \approx \exp\left(ikz+i\Phi \left(x,y\right)\right)\underset{m,n}{\sum }\beta_{m,n}\left(z\right)\\ & \;\times \exp(\frac{2\pi im}{p_{x}}\phi_{x}\left(x,y\right)+\frac{2\pi in}{p_{y}}\phi_{y}\left(x,y\right)), \end{array}$$

where

(17) $$\begin{array}{ll} & \phi_{x}\left(x,y\right)=x-\frac{i\lambda z}{2\pi }\frac{\partial \Phi }{\partial x}\left(x,y\right),\\ & \phi_{y}\left(x,y\right)=y-\frac{i\lambda z}{2\pi }\frac{\partial \Phi }{\partial y}\left(x,y\right). \end{array}$$

Note that a higher-order expansion is necessary when the phase shift is not small [25, 30, 31]. The intensity of the wave field is then given by

(18) $$\begin{array}{ll} I_{S}\left(x,y,z\right) & =\underset{m,n}{\sum }\rho_{m,n}\left(z\right)\\ & \;\times \exp(\frac{2\pi im}{p_{x}}\phi_{x}\left(x,y\right)+\frac{2\pi in}{p_{y}}\phi_{y}\left(x,y\right)). \end{array}$$

Let the complex transmittance function of G2 be

(19) $$T_{2}\left(x,y\right)=\underset{m,n}{\sum }b_{m,n}\exp\left(2\pi i\frac{mx}{p_{x}}\right)\exp(2\pi i\frac{ny}{p_{y}}),$$

with the same periods as those of the phase grating G1, a moiré pattern after the analyzer grating G2 is given by [22, 23]

(20) $$\begin{array}{ll} M_{k_{x},k_{y}}\left(x,y,z\right) & =I_{S}\left(x,y,z\right)\times T_{2}\left(x+\chi_{x},y+\chi_{y}\right)\\ & =\underset{m,n}{\sum }b_{m,n}\rho_{m,n}\left(z\right)\\ & \;\times \exp\left(\frac{2\pi im}{p_{x}}\left(\phi_{x}\left(x,y\right)+\chi_{x}\right)\right)\\ & \;\times \exp\left(\frac{2\pi in}{p_{y}}\left(\phi_{y}\left(x,y\right)+\chi_{y}\right)\right), \end{array}$$

where *χ_x_* = *k_x_* *p_x_*/*K_x_*, *k_x_* = 0, 1,…, *K_x_* − 1 and *χ_y_* = *k_y_* *p_y_*/*k_y_*, *k_y_* = 0, 1,…, *k_y_* − 1 are the scanned displacements of G2 in the *x* - and *y*-directions, respectively. The phase gradients can be reconstructed by the inverse discrete Fourier transform (DFT) as follows. By the inverse DFT,

(21) $$\begin{array}{ll} \eta_{m,n}\left(x,y\right) & =b_{m,n}\rho_{m,n}\left(z\right)\\ & \;\times \exp(\frac{2\pi im}{p_{x}}\phi_{x}\left(x,y\right)+\frac{2\pi in}{p_{y}}\phi_{y}\left(x,y\right))\\ & =\frac{1}{K_{x}K_{y}}[\overset{K_{x}-1}{\underset{k_{x}=0}{\sum }}\overset{K_{y}-1}{\underset{k_{y}=0}{\sum }}M_{k_{x},k_{y}}\left(x,y,z\right)\\ & \;\;\;\;\times \exp\left(\frac{2\pi ik_{x}m}{K_{x}}+\frac{2\pi ik_{y}n}{K_{y}})\right] \end{array}$$

if the grating coefficients *a_m,n_* and *b_m,n_* vanish rapidly when |*m*| and |*n*| are large compared to *K_x_* and *K_y_*. The coefficients *ρ_m,n_*(*z*) are of real values at the Talbot distance *Z_T_* hence

(22) $$\frac{2\pi m}{p_{x}}\phi_{x}\left(x,y\right)+\frac{2\pi n}{p_{y}}\phi_{y}\left(x,y\right)=\text{arg}[\eta_{m,n}\left(x,y\right)].$$

The lower-order terms such as *m* = ± 1 and *n* = ± 1 are typically used because their contributions are dominant in the measured fringes. Higher-order harmonics in (18) may cause error in the determination of the phase gradients. The error can be reduced by sophisticated fringe analyze methods with sufficiently large *K_x_* and *K_y_* [8, 21–23].

### 3. NUMERICAL EXPERIMENTS

We report numerical simulation results in this section. The grating transmission functions are chosen to be of the Ronchi ruling with the Fourier coefficients in (2) and (19) given by

(23) $$\text{sinc}\frac{m\pi }{2}\text{sinc}\frac{n\pi }{2}.$$

**Figure 2:**
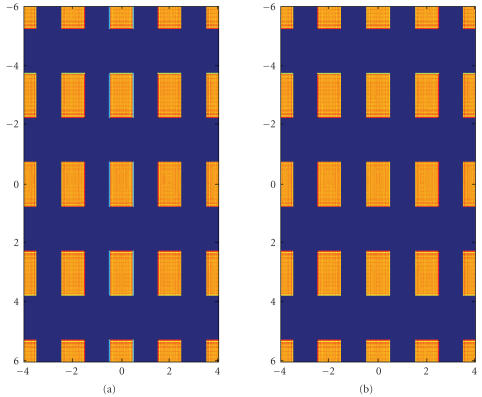
Intensity distribution for an incident coherent plane wave of energy of 40 keV behind the grating G1 of periods *p_x_* = 2 *μ*m and *p_y_* = 3 *μ*m The grating transmission function is assumed to be a Ronchi grating. The Talbot distance is *Z_T_* = 232cm. (a) The distribution of the grating transmission function. (b) The distribution of the wave intensity at the Talbot distance. The spatial unit is *μ*m.

The first experiment is to simulate the Talbot effect in Section 2.2.1. An incident coherent plane wave of energy of 40 keV is used. The grating periods are *p_x_* = 2*μ*m and *p_y_* = 3 *μ*m. We set *M* = 2 and *N* = 3 according to (7). The Talbot distance in (8) is *Z_T_* = 232cm. The result is shown in Figure 2.

The second experiment is to simulate the phase stepping measurement in Section 2.2.2 for phase objects. The X-ray energy is 40 keV and has a wavelength *λ* = 0.03nm. The parameters of the gratings G1 and G2 are *p_x_* = 4*μ*m and *p_y_* = 4*μ*m. The parameters are chosen according to those in [11]. The grating transmission function is assumed to be a Ronchi grating as in the previous experiment. The first Talbot distance is *Z_T_* = 1, 652 cm. When the grating periodicities are smaller, the Talbot distances will be smaller, too. The phase object is given by the following function:

(24) $$\Phi \left(x,y\right)=\Phi_{x}\left(x\right)\Phi_{y}(y),$$

where

(25) $$\begin{array}{ll} & \Phi_{x}\left(x\right)=\overset{S_{x}}{\underset{i=1}{\sum }}A_{x,i}\psi_{a_{x,i},b_{x,i}}\left(x\right),\\ & \Phi_{y}\left(y\right)=\overset{S_{y}}{\underset{j=1}{\sum }}A_{y,j}\psi_{a_{y,j},b_{y,j}}\left(y\right). \end{array}$$

*S_x_* and *S_y_* are the numbers of basis functions. *A_x,i_* and *A_y,j_* are the weights for the basis functions. *a_x,i_* and *a_y,j_* are the scale factors of the basis functions. *b_x,i_* and *b_y,j_* are the centers of the basis functions. The basis function *ψ_a,b_*(*t*) is as follows. Let *β*(*t*) be a nonnegative function with support in [− 1, 1] and let

(26) $$\alpha \left(t\right)=\int_{-\infty }^{t}\beta \left(s\right)ds.$$

For *a* > 0 and *b* ∈ *R*, let

(27) $$\begin{array}{ll} & \varphi_{a,b}\left(t\right)=\beta (\frac{x-b}{a}),\\ & \psi_{a,b}\left(t\right)=a\alpha \left(\frac{x-b}{a}\right). \end{array}$$

It follows that

(28) $$\begin{array}{ll} & \frac{\partial \Phi }{\partial x}\left(x,y\right)=\Phi_{y}\left(y\right)\overset{S_{x}}{\underset{i=1}{\sum }}A_{x,i}\varphi_{a_{x,i},b_{x,i}}\left(x\right),\\ & \frac{\partial \Phi }{\partial y}\left(x,y\right)=\Phi_{x}\left(x\right)\overset{S_{y}}{\underset{j=1}{\sum }}A_{y,j}\varphi_{a_{y,j},b_{y,j}}\left(y\right). \end{array}$$

Each *φ_a,b_* is of the support [*b* − *a*, *b*+*a*] . *a_x,i_* and *b_x,i_* are chosen such that the supports of the *φ_ax,i,bx,i_* are disjoint. *A_x,i_* < 10^−3^ are chosen such that the *ϕ_x_* and *ϕ_y_* in (17) are within the principle value interval [−*π*, *π*] to avoid the phase wrapping issue in finding the phase angle by (22). This magnitude 10^−3^ of *A_x,i_* is chosen to simulate weak phase objects. The *a_y,j_*, *b_y,j_* and *A_y,j_* are chosen in the same manner. We have used in our experiments the following basis functions:

(29) $$\begin{array}{ll} & \beta \left(t\right)=\left\{ \begin{array}{cc} 1-\left|x\right|, & \text{if}\left|x\right|<1,\\ 0, & \text{if}\left|x\right|<1, \end{array}\right.\\ & \beta \left(t\right)=\left\{ \begin{array}{cc} 1, & \text{if}\left|x\right|<1,\\ 0, & \text{if}\left|x\right|<1. \end{array}\right. \end{array}$$

We have run the simulation for both basis functions, different grating periodicities, and various stepping lengths. Consistent results are obtained. Representative results are shown in the following figures. Images are all sampled at the step 1000*λ* = 0.03 *μ*m in [−*p_x_*/2, *p_x_*/2] × [−*p_y_*/2, *p_y_*/2].

**Figure 3:**
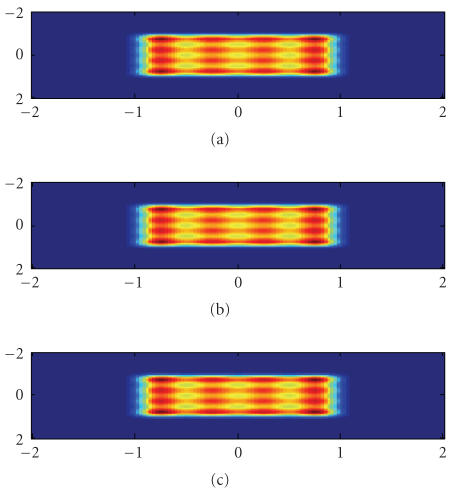
Wave intensity distributions after a phase object. (a) Right behind the grating G1; (b) At the first Talbot distance; (c) At the first Talbot distance by the first-order approximation. The spatial unit is *μ*m.

**Figure 4:**
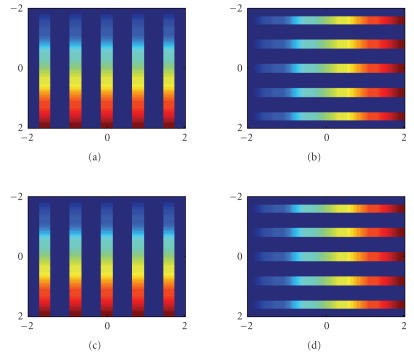
Distributions of phase gradients.The phase object is specified in (24) with *S_x_* = 5 and *S_y_* = 5. (a) The true *x*-gradient; (c) The measured *x*-gradient by phase stepping measurement. (b) and (d) are for the *y*-gradient. The spatial unit is *μ*m.

In Figure 3, the wave intensity distributions right behind the grating G1 are *z* = 0 *μ*m, and at the first Talbot distance are *z* = 1, 652cm by (14), and wave intensity at the first Talbot distance computed by the first-order approximation by (18) is presented. The result demonstrates that the first-order formula in (15) is a good approximation for weak phase objects in computing the downstream wave field. The parameters for the phase object in (24) are *S_x_* = 5 and *S_y_* = 5 The derivatives are presented in Figure 4. 

In Figure 4, the phase stepping technique is used to measure the derivatives of the phase objects. The same parameters as in Figure 3 are used. The phase steps are *χ_x_* = 17 and *χ_y_* = 17. The original distributions of gradients *Φ_x_* and *Φ_y_* and those reconstructed gradient distributions from (22) for *m* = 1, *n* = 0 and *m* = 0, *n* = 1 are presented in Figure 4. The errors in phase measurement compared to their exact values are smaller than the floating error at the orders 10^−17^ and 10^−9^% for the maximal absolute error and the maximal relative error, respectively.

### 4. DISCUSSIONS AND CONCLUSION

Talbot interferometry techniques can be applied for X-ray phase-contrast imaging using a polychromatic hospital grade source coupled with an amplitude grating to form a set of equidistant source wavelets. As a result, we can avoid using a synchrotron-radiation source or a microfocus X-ray tube. This may eventually lead to various grating-based X-ray phase-contrast imaging systems. Further research topics include better grating design, more accurate phase retrieval, optimized prototyping, and identification of the optimal X-ray energy balancing image resolution, contrast and dose, as well as in vivo testing in different applications such as mouse studies. We have demonstrated this technique at the first-order Talbot distance *Z_T_*. It is reported that the same technique works at the fractional Talbot distances in (11) with appropriate approximation for wave intensities under measurement.

Using the 2D grating-based phase-contrast imaging system, phase-contrast tomography is feasible. Tomography requires the phase deformation profile Φ(*x*, *y*, *θ*) which is the projection of the complex refractive index *δ*:

(30) $$\Phi \left(x,y,\theta \right)=\frac{2\pi }{\lambda }\int \delta \left(X,Y,z\right)dz,$$

where (*X*, *Y*, *z*) denotes a 3D position in an object to be reconstructed, and *θ* the angle between the *xy* and *XY* axes. To recover the projection in the form (30), we may calculate Φ(*x*, *y*, *θ*) by integrating its gradient along the *x*-direction or *y*-direction, or by a filtering technique as in [15, 18]. Then it is straightforward to reconstruct an underlying distribution of the refractive index *δ* using available CT methods. When the source emits a cone beam or beam shapes other than plane waves, G1 and G2 should have different periodicities and patterns. As there are theories on the Talbot effect available for spherical wave or Gaussian beams [25], the results in this manuscript can be extended to those cases. For example, in [32], the capillary plates with hexagonal patterns have been applied to phase-contrast imaging.

The results in the above are based on the paraxial approximation of the Fresnel integral for plane waves. The Talbot effect, based on a nonparaxial formulation, as well as spherical and Gaussian beams has been studied with similar results and can be applied for phase-contrast imaging with gratings [25]. The grating-based X-ray phase-contrast tomography can also be achieved using more complicated methods based on the Helmholtz equation, a fundamental governing equation [27]. In that case, the forward model would become more accurate at a much higher computational cost. Accordingly, we may have to use an iterative approach to reconstruct phase-contrast images from sufficiently many projections. This is a typical inverse coefficient problem of partial differential equations. The Born approximation can be used to find approximate solutions of the Helmholtz equation [33], with higher-order Born approximations used to improve the solution accuracy.

The main contribution of this paper is the use of 2D (chessboard) gratings so that a continuous operating hospital X-ray tube can be converted into a 2D array of coherent source wavelets, and truly 2D phase-contrast projections optimally formed. The use of 2D gratings instead of the state of the art 1D gratings has the major advantage of achieving nearly isotropic phase detection with fewer alignment problems. It is theoretically impossible to obtain both the *x*- and *y*-derivatives of a 2D phase object from 1D phase-stepping measurement along only one direction. We have simulated both 1D and 2D phase-stepping measurements and demonstrated this point. Two-axis scan with one 1D grating requires not only translations but also rotations, which means extra mechanical hardware. On the other hand, the 2D grating interferometry technique can reduce the mechanical rotations as compared to the 1D grating phase-stepping measurement. Hence, the 2D grating interferometry technique is more efficient than the 1D case. Up to date, no group has reported such a 2D-grating-based x-ray phase-contrast imaging system, which we are developing for biomedical applications. During the revision of this work, we noted the work [34], which was submitted on 2 August 2007, after our initial submission of this paper on 14 June 2007. The principal difference between our work and theirs is that our approach is interferometry-based while their *“coded-aperture approach is not an interferometric one, and it is therefore substantially different from the grating interferometer method”* [34].

In conclusion, we have described a 2D grating-based system and theory for superior phase retrieval and reported our numerical simulation results showing its feasibility. We are actively constructing the system for testing our algorithm with real data.

## Figures and Tables

**Figure 1:**
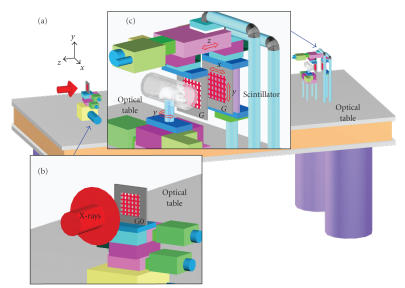
Schematics of our 2D grating-based phase-contrast image system. See the text for detailed configuration. Note that the scales of the grating features are exaggerated for illustration.

## References

[1] Momose A (2005). Recent advances in X-ray phase imaging. *Japanese Journal of Applied Physics, Part 1*.

[2] Als-Nielsen J, McMorrow D (2001). *Elements of Modern X-Ray Physics*.

[3] Momose A, Fukuda J (1995). Phase-contrast radiographs of nonstained rat cerebellar specimen. *Medical Physics*.

[4] Wilkins SW, Gureyev TE, Gao D, Pogany A, Stevenson AW (1996). Phase-contrast imaging using polychromatic hard X-rays. *Nature*.

[5] Wu X, Liu H (2007). Clarification of aspects in in-line phase-sensitive X-ray imaging. *Medical Physics*.

[6] Wu X, Liu H, Yan A (2005). X-ray phase-attenuation duality and phase retrieval. *Optics Letters*.

[7] Momose A, Kawamoto S, Koyama I, Hamaishi Y, Takai K, Suzuki Y (2003). Demonstration of X-ray Talbot interferometry. *Japanese Journal of Applied Physics, Part 2*.

[8] Momose A, Yashiro W, Takeda Y, Suzuki Y, Hattori T (2006). Phase tomography by X-ray Talbot interferometry for biological imaging. *Japanese Journal of Applied Physics, Part 1*.

[9] Weitkamp T, Diaz A, David C (2005). X-ray phase imaging with a grating interferometer. *Optics Express*.

[10] Weitkamp T, Nöhammer B, Diaz A, David C, Ziegler E (2005). X-ray wavefront analysis and optics characterization with a grating interferometer. *Applied Physics Letters*.

[11] Pfeiffer F, Weitkamp T, Bunk O, David C (2006). Phase retrieval and differential phase-contrast imaging with low-brilliance X-ray sources. *Nature Physics*.

[12] Talbot F (1836). Facts relating to optical science no. IV. *The London and Edinburgh Philosophical Magzine and Journal of Science*.

[13] Lohmann AW, Silva DE (1971). An interferometer based on the Talbot effect. *Optics Communications*.

[14] Yokozeki S, Suzuki T (1971). Shearing interferometer using grating as the beam splitter. *Applied Optics*.

[15] Kottler C, David C, Pfeiffer F, Bunk O (2007). A two-directional approach for grating based differential phase contrast imaging using hard X-rays. *Optics Express*.

[16] Weitkamp T, David C, Kottler C, Bunk O, Pfeiffer F, Bonse U Tomography with grating interferometers at low-brilliance sources.

[17] Koyama I, Momose A, Jin WU, Lwin TT, Takeda T (2005). Biological imaging by X-ray phase tomography using diffraction-enhanced imaging. *Japanese Journal of Applied Physics, Part 1*.

[18] Pfeiffer F, Kottler C, Bunk O, David C (2007). Hard X-ray phase tomography with low-brilliance sources. *Physical Review Letters*.

[19] Pfeiffer F, Grünzweig C, Bunk O, Frei G, Lehmann E, David C (2006). Neutron phase imaging and tomography. *Physical Review Letters*.

[20] Creath K, Wolf E (1988). Phase measurement interferometry techniques. *Progress in Optics XXVI*.

[21] Surrel Y (1996). Design of algorithms for phase measurements by the use of phase stepping. *Applied Optics*.

[22] Patorski K (1993). *Handbook of the Moiré Fringe Technique*.

[23] Momose A, Yashiro W, Moritake M, Bonse U Biomedical imaging by Talbot-type X-ray phase tomography.

[24] Winthrop JT, Worthington CR (1965). Theory of fresnel images. I. Plane periodic objects in monochromatic light. *Journal of the Optical Society of America*.

[25] Patorski K, Wolf E (1989). The self-imaging phenomenon and its applications. *Progress in Optics XXVII*.

[26] Arrizón V, Rojo-Velázquez G (2001). Fractional Talbot field of finite gratings: compact analytical formulation. *Journal of the Optical Society of America A*.

[27] Born M, Wolf E, Bhatia AB (1999). *Principles of Optics: Electromagnetic Theory of Propagation, Interference and Diffraction of Light*.

[28] Lohmann AW, Knuppertz H, Jahns J (2005). Fractional montgomery effect: a self-imaging phenomenon. *Journal of the Optical Society of America A*.

[29] Guigay JP (1971). On fresnel diffraction by one-dimensional periodic objects, with application to structure determination of phase
objects. *Journal of Modern Optics*.

[30] Salama NH, Patrignani D, De Pasquale L, Sicre EE (1999). Wavefront sensor using the Talbot effect. *Optics and Laser Technology*.

[31] Siegel Ch, Loewenthal F, Balmer JE (2001). A wavefront sensor based on the fractional Talbot effect. *Optics Communications*.

[32] Momose A, Kawamot S (2006). X-ray Talbot interferometry with capillary plates. *Japanese Journal of Applied Physics, Part 1*.

[33] Jonas P, Louis AK (2004). Phase contrast tomography using holographic measurements. *Inverse Problems*.

[34] Olivo A, Speller R (2007). Modelling of a novel x-ray phase contrast imaging technique based on coded apertures. *Physics in Medicine and Biology*.

